# Supplementation of Foals with a *Saccharomyces cerevisiae* Fermentation Product Alters the Early Response to Vaccination

**DOI:** 10.3390/ani14060960

**Published:** 2024-03-20

**Authors:** Eva Ronja Terpeluk, Jana Schäfer, Christa Finkler-Schade, Hans-Joachim Schuberth

**Affiliations:** 1 Institute for Immunology, University of Veterinary Medicine Foundation, Bünteweg 2, 30559 Hannover, Germany; eva.terpeluk@tiho-hannover.de; 2 Schäfer Horse Breeding, 38159 Vechelde, Germany; jana_schaefer@outlook.com; 3 Schade & Partner, 27283 Verden, Germany; cs@schadeundpartner.de

**Keywords:** *Saccharomyces cerevisiae*, SCFP, prebiotics, postbiotics, diarrhea, vaccination, horses, foals

## Abstract

**Simple Summary:**

Many foals suffer from foal heat diarrhea in the first weeks of life. The objective of this study was to determine whether feeding a *Saccharomyces cerevisiae* fermentation product affects the occurrence of early foal diarrhea, and whether this feeding during the first four weeks of life has a long-term influence on the immediate response to a parenteral vaccination at the age of 6 to 9 months. Newborn foals were fed a supplement or a placebo for 29 days and were vaccinated against influenza and tetanus in the age of 6–9 months. Blood leukocyte subset numbers were determined before and 24 h after vaccination. Leukocyte numbers and blood cell composition did not differ significantly between the supplemented group and the placebo group on the day of vaccination, whereas only supplemented foals showed significant changes in the number of circulating leukocyte populations after vaccination. Thus, the pre- and postbiotic supplementation had far-reaching consequences for innate immune mechanisms later in life.

**Abstract:**

Feed supplements supporting animal welfare and performance are becoming increasingly important. Immunomodulatory effects of such products have been observed in many species. The aim of this study was to analyze whether food supplementation with a *Saccharomyces cerevisiae* fermentation product (SCFP) affects the occurrence of foal diarrhea in early life, and whether the SCFP feeding has an impact on the immediate response to a parenteral vaccination at the age of 6–9 months. Eleven foals received the SCFP (OLI) and eleven foals were fed a placebo (PLA) for 29 days. Growth, diarrhea, and diarrhea severity were observed until day 30. After weaning, at the age of 6–9 months, foals were vaccinated parenterally against influenza and tetanus. The supplementation had no statistically significant effect on diarrhea duration and severity. On the day of vaccination, PLA and OLI foals did not differ significantly regarding numbers of circulating blood leukocyte subsets. However, the response to vaccination differed significantly between OLI and PLA foals. In OLI foals, the numbers of the major leukocyte fractions (granulocytes, lymphocytes, monocytes, CD4+ T cells, CD8+ T cells, CD21+ B cells, and MHC-II+/CD21− cells) increased significantly 24 h after vaccination but remained unchanged in PLA foals. The observed results suggest that early life supplementation with an SCFP may affect the early immune response to an initial vaccination.

## 1. Introduction

The most common causes of early foal losses are respiratory and gastrointestinal diseases [1]. Between days 5 and 15 after parturition, when the dam’s estrus is expected, a large proportion of foals experience foal heat diarrhea (FHD). The contemporaneous appearance of the early diarrhea with the mare’s first estrus period after foaling gave the foal heat diarrhea its name. FHD is probably the result of physiologic gastrointestinal changes [2,3]. This includes dynamic changes within the foals’ developing gut microbiome from birth until approximately 60 days of age. FHD incidences range from 19 to 80% [2,4,5], due to different diarrhea definitions and other management factors. The diarrhea is often described as a self-limiting process lasting 3 to 4 days without signs of systemic disease or inflammation [2,6,7]. The long-term health consequences of FHD are unknown. However, compared to healthy foals, a lower fecal bacterial microbial richness and diversity was reported in diarrheic foals [3,4] and fecal samples of non-diarrheic foals were enriched with bacteria involved in gastrointestinal health [3,4]. 

In humans, the influence of an early life gut microbiota dysbiosis due to diarrhea incidences or the use of antibiotics is recognized as a risk factor for the development of several immune-mediated diseases and metabolic disorders later in life [8,9]. This suggests that early life disturbances in the gut may also have consequences for initial immune mechanisms after contact with pathogens and after vaccinations [10].

Attempts to ameliorate foal diarrhea included the supplementation with distinct microorganisms (*Bacillus cereus* var. *toyoi*), which had no recognizable effect on foals’ health or diarrhea events [11]. Feeding neonatal foals with probiotic strains of *Lactobacillus rhamnosus* and *Enterococcus faecium* has been found to be associated with a higher diarrhea incidence and longer periods compared to placebo-fed groups [1,12,13], although other studies reported on lower diarrhea incidence and increased weight gains after feeding probiotic strains or mixtures of strains [14,15,16].

*Saccharomyces cerevisiae* fermentation products are used as supplements in different species to improve health, growth, feed efficiency, and productivity and to reduce diarrhea [17,18,19,20,21]. Supplemented weanling pigs showed enhanced growth performance and a better protection against the K88 ETEC infection (reduced diarrhea and intestinal damage) [20]. This is consistent with the observation of a less enteric pathogen colonization after an ETEC challenge of supplemented piglets [18]. SCFP-supplemented calves, on the other hand, did not show enhanced food intake or body weight, or a reduced diarrhea incidence [22].

Many studies have demonstrated the immunomodulatory effects of SCFP supplementation [23,24,25,26]. SCFP-supplemented weaned pigs showed an increased pro-inflammatory cytokine response to a LPS challenge compared to a control group [23]. In dairy calves, SFCP supplementation resulted in an increased acute immune response (increased TNF-α, glucose, and respiration rate) compared with a control group after a lipopolysaccharide challenge [27]. SCFP-supplemented calves challenged with bovine respiratory syncytial virus showed altered systemic and mucosal immune responses and a lower viral load in the lungs [26]. Further, altered immune responses after a vaccine challenge has been reported for broilers [24,28], horses [29], and bovines [30].

In horses, SCFPs were used to ameliorate exercise-induced stress responses in quarter horse yearlings. An eight-week supplementation period resulted in a quicker return to pre-exercise cortisol values, and serum amyloid A (SAA) level did not increase after the stressful event [31]. Previous studies demonstrated an altered vaccination response in SCFP-supplemented racehorses. On the day of vaccination, the two supplementary groups did not differ in blood composition. However, 24 h after vaccination, different changes in blood leukocyte composition and reticulocyte fractions between those groups were determined, which indicates that SCFP supplementation modulates initial vaccination-induced responses [29,32].

The objective of this study was to analyze whether food supplementation with a *Saccharomyces cerevisiae* fermentation product affects the occurrence of foal heat diarrhea during the first weeks of life, and whether SCFP feeding has an impact on the immediate response to a parenteral vaccination at the age of 6–9 months.

## 2. Materials and Methods

### 2.1. Animals and Diet

**Figure 1:**
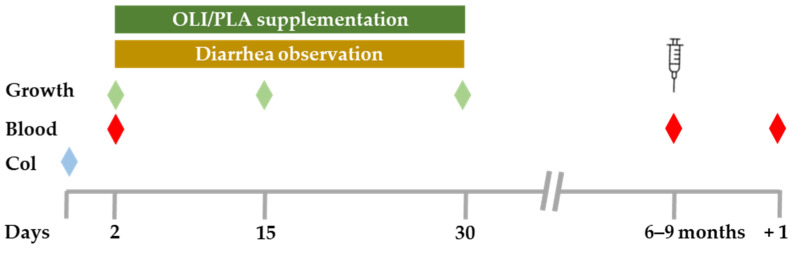
Experiment design. Twenty-two foals were randomly assigned to two supplementation groups (OLI, *n* = 11; PLA, *n* = 11). The SCFP (OLI) and the placebo (PLA) were supplemented between day 2 and day 30. Occurrence of diarrhea and fecal scores were determined daily between day 2 and day 30. Height and body weight were determined on days 2, 15, and 30. Colostrum (Col) samples were taken within 3 h of delivery. Blood samples were collected on day 2, on the day of vaccination (age of 6–9 months), and 24 h after vaccination (+1).

Twenty-two warmblood foals from a farm in Lower Saxony were included in the study. The experiment was approved by the state of Lower Saxony, Germany, in accordance with § 8(1) of the Animal Protection Act in conjunction with § 33 of the Animal Protection Experimental Animal Regulations (File number: 33.19-42502-04-22-00156). All foals born in the 2022 foaling season on this farm were used in the trial. Farm staff who fed the supplement or the placebo and who assessed the foals and the diarrhea were blinded. All mares were stabled in the same facility in individual boxes. After the morning feeding mares and their foals were taken in groups to the pasture, paddock, or indoor arena, depending on the weather conditions. After the supplementation and diarrhea observation period, the mares and their foals were moved to permanent pasture. Mares were handled the same way regarding paddock/grazing times, feeding, and feeding times. Foals were handled the same way in terms of feeding, handling, weaning, and, as far as possible, regarding turnout times. Due to the fact that the first foals were born in January and the last ones in June, an identical turnout handling was not possible for all foals. The mares were allocated to two groups to ensure the horses were equally distributed regarding age and parity (Table S1). The foals were grouped according to this classification (OLI: 10 fillies, 1 colt; PLA: 9 fillies, 2 colts). Mares and foals always had access to water. Mares received high quality hay, oats, barley, and minerals twice a day in approximately the same quantity. Foals were fed the same food after starting to ingest solid food. The supplements were provided during the morning feeding by hand from a bowl to ensure the complete uptake of the ratio by each foal. If a foal did not eat its portion, the supplement was mixed with tap water and administered with an oral syringe. The foals received 10 g dry powder of either the SCFP supplement (OLI, *n* = 11) or the placebo (PLA, *n* = 11). Supplementation started on day 2 and ended on day 30 (Figure 1). One PLA foal died three days after birth. One OLI foal died during the trial, suffering colic before weaning. Foal number 1 showed itching on days 24 and 25, which subsided without medication. Foal number 5 had musculoskeletal problems at day 14 and received a four-day Meloxicam treatment (twice a day). Foal number 6 showed a swollen navel on day 3 and an elevated rectal body temperature (39 °C). This foal received Ceftiofur 4 mg/kg twice a day i.m. and Meloxicam. Six days later, a urachal fistula was surgically removed. Post operation, the foal received Trimethoprim twice a day for 7 days and Meloxicam. Foal number 15 appeared on day 2 with a contracted tendon and was treated once with 30.000 IE Oxytetracyclin (Ursocyclin^®^ 10% Pro Inj, Serumwerk Bernburg AG, Bernburg, Germany) i.v. Foal number 22, suffering musculoskeletal problems at day 1, received Ursocyclin^®^ 10% (30 mL) for 3 days.

Mares were dewormed with Ivermectin (Bimectin Paste 18.7 mg/g, Bimeda^®^, Dublin, Ireland) the day of parturition, in spring with Pyrantel (152.2 mg/g, CP-Pharma Handelsgesellschafft mbH, Burgdorf, Germany) and in autumn after weaning with Moxidectin + Praziquantel (Equest^®^ Pramox 19.5 mg/g + 121.7 mg/g, Zoetis Deutschland GmbH, Berlin, Germany). Foals at the age of six weeks were dewormed with Pyrantel and every six weeks again with Pyrantel. After vaccination, all foals were dewormed with Ivermectin.

Ingredients of OLI and PLA are listed in Table 1. The used SCFP (Olimond BB; BB horses GmbH & Co. KG, Kreuztal, Germany) is a freely available feed supplement in EU member states with the following analytical constituents and contents: 21.10% crude protein, 1.90% crude fat, 19.20% crude fiber, 9.20% crude ash, and 0.00% sodium.

### 2.2. Growth and Diarrhea Observation 

For growth observation, all foals were weighed and height was measured on days 2, 15 and 30 (Figure 1). Weight detection was performed using a floor scale (model SBS-PS-300, Steinberg^®^ systems, Berlin, Germany). Height was determined at the withers using an aluminum measuring stick (180 cm). Furthermore, on days 2, 15 and 30, health conditions (respiration rate and heart rate) were checked and rectal body temperature (clinical thermometer; SCALA Electronic GmbH, Stahnsdorf, Germany), nutritional condition, coat, character of feces, conformation of limbs, and behavior were documented by an experienced owner (farm veterinarian) (Table S4). Diarrhea events were observed daily during the morning routine by the farm veterinarian for 29 days, starting on day 2. Noticeable changes throughout the day were documented by farm employees. Diarrhea classification (Table S2) was based on the scoring system presented by Ströbel et al. [1]. Feces with a score of 1 or 2 were classified as diarrhea. Feces scores (1–4) from all 29 days were transformed into severity points and added up to obtain the diarrhea severity. Foals were divided into two diarrhea groups, representing mild vs. severe courses. Foals suffering 0–5 days from diarrhea were classified in diarrhea group 1 (DG 1). One foal showed no signs of diarrhea (0 days) and was included in this group with mild diarrhea. Foals with 6–8 diarrheic days were assigned to diarrhea group 2 (DG 2). 

### 2.3. Colostrum and Blood Sample Collection

Colostrum samples were taken within 3 h of parturition to determine the colostrum refractometry index using a BRIX refractometer (RHB-190OE, HHTEC^®^, Hong Han GmbH, Heidelberg, Germany). The first streams of colostrum from each side were discarded and the colostrum samples were then collected from both sides in a sterile 15 mL centrifuge tube (article 02-502–3001, nerbe plus GmbH & Co. KG, Winsen/Luhe, Germany). According to good veterinary practice, all foals were clinically checked immediately before blood sampling, including examination of conjunctiva, oral mucosa, lymph nodes, and measuring the rectal body temperature using a clinical thermometer (SCALA Electronic GmbH, Stahnsdorf, Germany). On day 2, on the day of vaccination (age of 6–9 months), and 24 h after vaccination, blood was collected from the jugular vein via a puncture with a disposable needle (20 G × 1½, Sterican^®^, B. Braun SE, Melsungen, Germany) and using a 10 mL syringe (Injekt^®^, B. Braun SE, Melsungen, Germany) (Figure 1). On day 2, blood was collected to determine the total serum protein (TP), using a refractometer (RHC-300, HHTEC^®^, Hong Han GmbH, Heidelberg, Germany). In cases of TP values < 5.5 g/dL (3 foals), a rapid IgG concentration test (Fassisi Equine IgG-test, Göttingen, Germany) was performed. None of these foals suffered from a failure of passive transfer. Blood samples taken before and 24 h after vaccination were taken via venipuncture into BD Vacutainer^®^ Sodium Heparin Tubes (Medicalis Medizintechnologie, Hannover, Germany). Analysis of the samples took place within 24 h at the Institute for Immunology, University of Veterinary Medicine Foundation, Hannover, Germany.

### 2.4. Determination of Total Leukocyte Counts and Leukocyte Subpopulations

**Figure 2:**
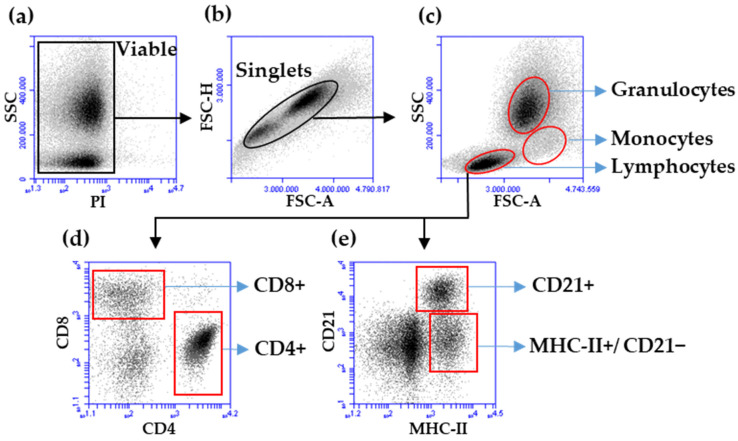
Gating strategy for determination of leukocyte fractions (**a**–**e**) using density plots. (**a**) Viable, propidium iodide-negative leukocytes were identified according to their morphology and in a PI/SSC density plot. (**b**) Single cells were detected among viable cells in an FSC-A/FSC-H density plot. (**c**) Regions identifying viable, single granulocytes, monocytes, and lymphocytes in a FSC-A/SSC density plot. Identification of CD4+ and CD8+ T cells among lymphoid cells after a staining with directly labelled CD4- and CD8-specific antibodies (**d**) or CD21+ B cells and MHC-II+CD21− lymphocytes after staining with directly labelled MHC-II− and CD21-specific antibodies (**e**).

Whole heparinized blood (20 μL) was mixed with 180 μL Turk’s solution, and 20 μL were applied to a Bürker chamber. Leukocytes were counted manually using a microscope (Nikon microscope ECLIPSE 80i, Nikon Europe B.V., Amstelveen, The Netherlands). The fractions of major leukocyte subpopulations (granulocytes, monocytes and lymphocytes) were determined using flow cytometry (BD Accuri™ C6 Flow Cytometer, Becton Dickinson Inc., Holdrege, NE, USA). For this, 2 mL whole heparinized blood was mixed with 20 mL distilled water (DW) for 30 s, followed by the addition of 20 mL double-concentrated phosphate-buffered saline (2× PBS). After centrifugation (500× *g*, 10 min, 8 °C), the supernatant was discharged and the cell pellet suspended. The hypotonic lysis step was repeated. The cell pellet was suspended in 2 mL PBS and mixed for 30 s. To 200 μL cell suspension, 200 μL sterile filtered PBS (0.4 μg/mL propidium iodide) (PBS-PI) was added, mixed for 20 s, and the suspension was analyzed flow cytometrically (20,000 events) (Figure 2). Viable granulocytes, monocytes, and lymphocytes were identified in forward (FSC)/side scatter (SSC) density plots according to their characteristic FSC/SSC profiles (Figure 2a–c). The percentages were multiplied by the total leukocyte counts to obtain the total numbers of the subpopulations. The reduction of the sample size was due to severe technical problems (clumping) after hypotonic lysis of heparinized blood samples after vaccination. This turned out to be a general problem with foal blood (as opposed to blood from adult horses). 

### 2.5. Membrane Immunofluorescence

200 μL leukocyte cell suspension/well were pipetted in a 96-well plate (96-well round base micro test plate, SARSTEDT AG & Co. KG, Nümbrecht, Germany) and centrifuged (402× *g*, 5 min, 8 °C). The supernatant was discharged and the cell pellet was suspended. Three separate sets were labelled with monoclonal antibody (mAK) combinations (30 μL). Set 1 contained anti-eqCD4-fluorescein isothiocyanate (FITC) (MCA1078F, IgG1 0.1 mg/mL, 1:100, Bio-Rad Laboratories GmbH, Feldkirchen, Germany) and anti-eqCD8-RPE (MCA1080PE, IgG2a 100 tests/mL, 1:10, Bio-Rad Laboratories GmbH). Set 2 contained anti-eqCD4-FITC, anti-canine CD21-AlexaFluor^®^647 (cross-reactive with the equine orthologue; MCA1781A647, IgG1 0.05 mg/mL, 1:200, Bio-Rad Laboratories GmbH), and anti-eqMHC-II-RPE (MCA1085PE, IgG1, 100 tests, 1:10, Bio-Rad Laboratories GmbH). Set 3 included isotype controls (MCA1209F, IgG1-FITC, Bio-Rad Laboratories GmbH; MCA929PE, IgG2a-PE, Bio-Rad Laboratories GmbH) of equal concentrations to ensure that no nonspecific reaction of the applied monoclonal antibodies with equine leukocytes occurred. Plates were incubated for 30 min on ice. After incubation, cells were washed twice with 200 μL membrane immunofluorescence (MIF) buffer (PBS, bovine serum albumin 5.0 g/L, sodium azide 0.1 g/L) for 5 min at 402× *g*. Cell pellets were suspended in 200 μL MIF buffer, 200 μL PBS-PI was added and cells were analyzed flow cytometrically after gating on viable lymphoid cells (Figure 2). CD4+ T cells, CD8+ T cells, CD21+ B cells, and MHC-II+/CD21− lymphoid cells were multiplied with the absolute number of lymphoid cells/mL blood. Fluorescence compensations were applied after flow cytometric acquisition in the BD Acurri C6 plus software version 1.0.34.1 (Figure S3).

### 2.6. Vaccination

At the age of 6–9 months, the foals were allocated to two weaning/vaccination groups according to age, general condition, and the assessment of the veterinarian on site (Table S3). Before vaccination, the animals were clinically examined and the rectal body temperature was measured to ensure that all foals were in a healthy condition. Foals were vaccinated with a commercial vaccine against influenza and tetanus PROTEQFLU™-TE (1 mL Influenza A/eq/Ohio/03 [H3N8] recombinant of Canarypox virus (strain vCP2242), Influenza A/eq/Richmond/1/07 [H3N8] recombinant of Canarypox virus (strain vCP3011) and *Clostridium tetani* toxoid ≥ 30 I.U. with carbomer as the adjuvant, Boehringer-Ingelheim Vetmedica GmbH, Ingelheim am Rhein, Germany). The vaccine was administered intramuscularly in the left breast using a 3 mL syringe (Omnifix^®^ Luer Solo, B. Braun SE, Melsungen, Germany) with a disposable needle (22G × 1¼, Sterican^®^, B. Braun SE, Melsungen, Germany) after cleaning according to standard practice. Twenty-four hours after vaccination the foals were checked again for vaccination side effects such as swollen injection site, pain, fever, or reduced general state of health.

### 2.7. Statistical Analysis

The number of animals is expressed as n. Data were visualized using GraphPad Prism v9.0.0 (for Windows 64-bit, GraphPad Software, San Diego, CA, USA). Data were analyzed using the SAS^®^ statistical software, version 9.4M7 with SAS Studio Enterprise, version 3.8.2 (SAS Institute Inc., Cary, NC, USA). Normal distribution was tested using the Shapiro-Wilk test and assumed if *p* > 0.05. In case of normal distribution, the equality of variances was checked (folded F test). A *p* value > 0.05 showed equal variances. Data with equal variances were tested using an unpaired pooled *t*-test: Values from blood samples (OLI vs. PLA; diarrhea group 1 (DG1) vs. diarrhea group 2 (DG2)) before and after vaccination. For unequal variances, the Welch-Satterthwaite *t*-test was used. In case of non-normal distribution, the Wilcoxon two-sample test was performed. Comparisons within a group regarding different times (before and after vaccination) were determined with a paired *t*-test (no normal distribution tested due to the small sample size of *n* = 5). Data, including height and weight at three different time points (d2, d15, d30), were tested using mixed models with supplement groups and days and the combination as fixed effects, height or weight as dependent variables, and the foal’s ID as subject for identification. Days were entered as repeated measurements (same animal; dependent measurement). Differences between groups for each individual time point and individual parameter (Brix, TP, diarrhea days and diarrhea severity) were examined with the Wilcoxon two-sample test. The Fisher’s exact test was used to determine whether there were differences in the number of foals in DG1 or DG2, when the foals were fed either the supplement or the placebo. *p* values ≤ 0.05 showed a statistical significance, and *p* values between 0.1 and 0.05 were defined as a trend.

## 3. Results

### 3.1. SCFP Feeding Did Not Affect the Growth of the Foals

Foals of both groups were born to mares of similar age and parity number (Table S1) and ingested colostrum of similar quality. The average Brix value of ingested colostrum ranged between 17% and 32% (median 23.5%, OLI) and 22% and 33% (median 25.0% PLA) (Table 2). On day 2 after colostrum intake, PLA foals presented with slightly but insignificantly higher serum protein values compared to OLI foals (Table 2). The growth of the foals (body height and weight) during the 29-day observation period showed no difference between the groups (Table 2).

### 3.2. SCFP Feeding Did Not Alter the Duration and the Severity of Foal Heat Diarrhea 

On average, OLI foals suffered from diarrhea with watery or thin mushy feces 2.0 ± 4.5 days compared to PLA foals (6.0 ± 5.5 days) (*p* = 0.1347, Table 3). OLI foals presented with a lower, but not statistically significant, overall diarrhea severity (8.0 ± 16.0) compared to PLA foals (23.5 ± 21.25) (*p* = 0.1288, Table 3). The grouping of foals into diarrhea groups (DG1 and DG2) revealed no statistically significant differences (Table 4; *p* = 0.1984).

### 3.3. SCFP Feeding Altered the Immediate Response to Vaccination

**Figure 3:**
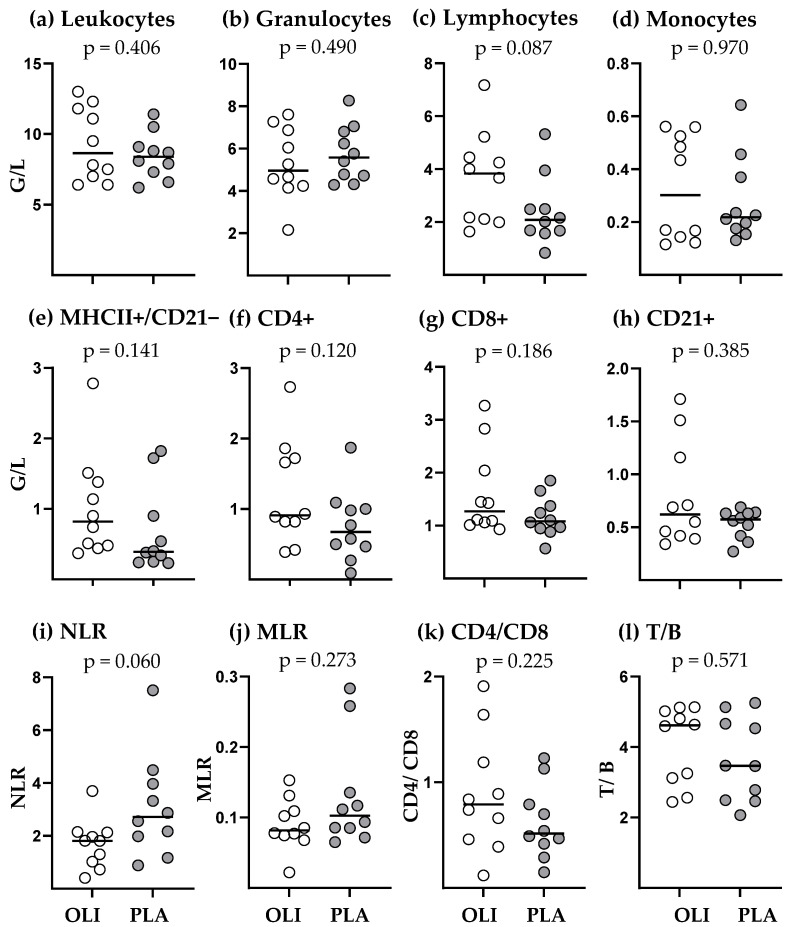
Numbers of major leukocyte populations, leukocyte subpopulations, and ratios between leukocytes in foal blood before vaccination at the age of 6–9 months after weaning (OLI, *n* = 10; PLA, *n* = 10). Total leukocyte numbers (**a**) were determined in a counting chamber and were used to calculate absolute numbers (G/L, giga/liter) of leukocyte subpopulations (**b**–**h**) after flow cytometric measurement of their fraction among leukocytes. (**e**): MHC-II+ and CD21− lymphocytes; (**f**): CD4+ T cells; (**g**): CD8+ T cells; (**h**): CD21+ B cells. (**i**–**l**): Ratios between neutrophils and lymphocytes ((**i**): NLR), monocytes and lymphocytes ((**j**): MLR), CD4+ and CD8+ T cells ((**k**): CD4+/CD8+), T (sum of CD4+ and CD8+ T cells) and B cells ((**l**): T/B). *p* values were determined by unpaired *t*-test or Wilcoxon two-sample test.

**Figure 4:**
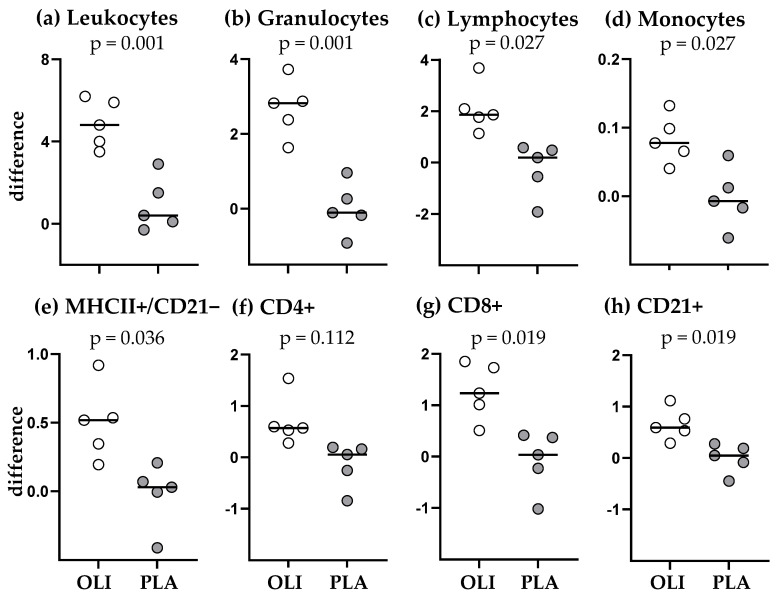
Changes in numbers of circulating major leukocyte populations and leukocyte subpopulations within 24 h of vaccination of foals (OLI, *n* = 5; PLA, *n* = 5). Total leukocyte numbers (**a**) were determined in a counting chamber and were used to calculate absolute numbers of leukocyte subpopulations (**b**–**h**) after flow cytometric measurement of their fraction among leukocytes. The values show the difference between the absolute numbers determined after and before vaccination ((**e**): MHC-II+ and CD21− lymphocytes; (**f**): CD4+ T cells; (**g**): CD8+ T cells; (**h**): CD21+ B cells). Difference: absolute cell number (giga/liter blood) after vaccination minus the absolute cell number (giga/liter blood) before vaccination. *p* values were determined by unpaired *t*-test or Wilcoxon two-sample test.

On the day of vaccination at the age of 6–9 months, OLI and PLA foals showed insignificantly different numbers of circulating leukocytes (Figure 3a). The same was observed for the major myeloid cell types (granulocytes, monocytes) (Figure 3b,d), whereas PLA foals had in tendency lower numbers of circulating lymphocytes (2.42 G/L ± 1.31; mean ± SD) compared to OLI foals (3.67 G/L ± 1.75; mean ± SD) (Figure 3c, *p* = 0.087). Conversely, the Neutrophil/lymphocyte ratio (NLR) was in tendency higher in the blood of PLA foals (3.093 ± 1.92; mean ± SD) compared to OLI foals (1.704 ± 0.92; mean ± SD) (Figure 3i; *p* = 0.060). The numbers of other blood cell subpopulations and ratios between cell types did not differ significantly between OLI and PLA foals. Twenty-four hours after vaccination, the changes in numbers of blood leukocytes and their subpopulations showed marked differences between OLI and PLA foals (Figure 4). Whereas in OLI foals the numbers of leukocytes, granulocytes, monocytes, lymphocytes, MHC-II+/CD21− cells, CD8+ T cells, and CD21+ B cells increased after vaccination (positive difference, Figure 4b–h), they remained rather unaffected in PLA foals (difference around zero). The differences in vaccination-induced cell number changes were significant for leukocytes (*p* = 0.001), granulocytes (*p* = 0.001), monocytes (*p* = 0.027), lymphocytes (*p* = 0.027), MHC-II+/CD21− (*p* = 0.036), CD8+ T cells (*p* = 0.019), and CD21+ B cells (*p* = 0.019).

**Figure 5:**
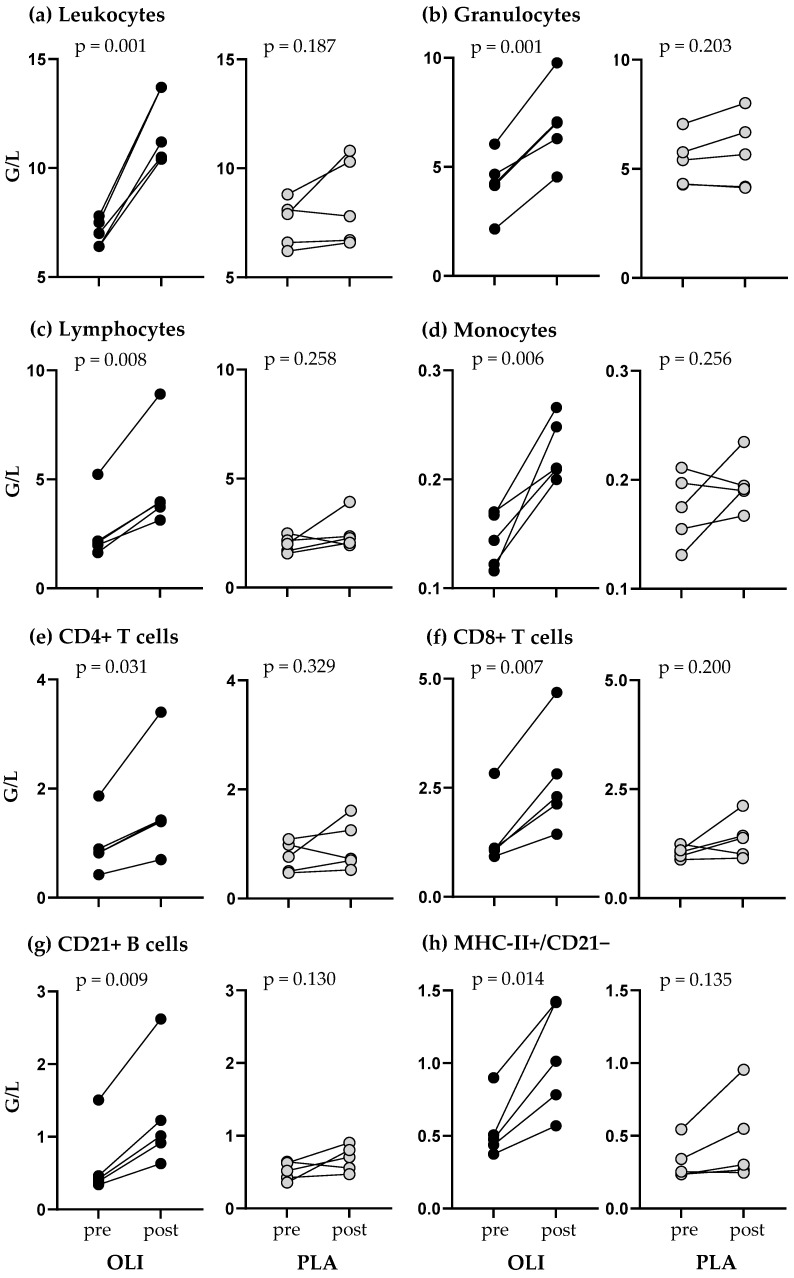
Group-specific changes of blood leukocyte numbers. Vaccination-induced changes in OLI (*n* = 5) and PLA foals (*n* = 5) in terms of total leukocyte numbers and numbers of leukocyte subpopulations (G/L, giga/liter) were determined on the day of vaccination (pre) and 24 h after vaccination (post). Total leukocyte numbers (**a**) were determined in a counting chamber and were used to calculate absolute numbers of leukocyte subpopulations (**b**–**h**) after flow cytometric measurement of their fraction among leukocytes ((**b**): granulocytes; (**c**): lymphocytes; (**d**): monocytes; (**e**): CD4+ T cells; (**f**): CD8+ T cells; (**g**): CD21+ B cells; (**h**): MHC-II+/CD21− lymphocytes). *p* values describe significances within a group and were calculated using a paired *t*-test.

Comparing the vaccination response within a group in terms of individual pre- versus post-vaccination values, it is noticeable that leukocyte counts and the major leukocyte fractions of all five OLI foals increased significantly with *p* values in the range of 0.001–0.031, whereas they remained the same or increased slightly in PLA foals without statistical significance (*p* = 0.130–0.329) (Figure 5).

## 4. Discussion

A rather high proportion of newborn foals, up to 80%, develop a condition known as foal heat diarrhea (FHD) in the first few weeks of their lives [2,4,5]. In our study, only one of 22 foals did not develop any signs of diarrhea during the first 30 days of life, whereas 21 foals suffered for various number of days from diarrhea. An insufficient feeding of colostrum could be ruled out since no foal showed up with reduced serum IgG values 1 day after colostrum uptake. 

Food supplements based on products of *Saccharomyces cerevisiae* are commonly fed to animals to prevent or diminish diarrhea [33]. We therefore addressed the question of whether SCFP supplementation during the first 30 days of a newborn foal’s life reduces the occurrence and severity of FHD and whether SCFP supplementation might have a long-term influence on the response of foals to their first vaccination after weaning.

In our study, SCFP supplementation had no statistically significant effect on the duration and severity of FHD (Table 3, *p* = 0.1984). Although the duration and severity of FHD was numerically lower in SCFP-supplemented foals, our study may have been underpowered to detect significant differences between SCFP-supplemented and placebo-fed foals. Further studies with a larger number of foals are necessary to draw conclusions on the effects of SCFPs on FHD prevalence and severity. Our results are in contrast to a comparable low-animal-number experiment, in which 4 of 10 placebo-fed foals developed diarrhea whereas none of the foals fed a probiotic *Enterococcus faecalis* strain (CECT7121) developed FHD [16]. Different diarrhea definitions and the implementation of diarrhea observation may be the main reasons for the different diarrhea incidence ranges. 

Whether the environment in which the foals were raised was not ideal and thus supported the onset of FHD [4,34] was not addressed. Another commonly stated factor for the occurrence of FHD is the increased susceptibility to pathogens after uptake of insufficient amounts of colostrum and/or its low quality [34,35]. This could be ruled out since the Brix values of fed colostrum did not differ significantly between PLA and OLI foals and insignificant differences in the total protein content of blood serum after colostrum uptake indicated that foals of both groups had been fed similar colostrum amounts (Table 2). 

The precise mechanisms behind a probable SCFP-mediated diarrhea reduction have not yet ben elucidated and were not analyzed in the present study. In a controlled study with piglets, feeding with an SCFP reduced the number of ETEC attached to the ileal mucosa [18]. Varying effects of SCFP feeding on the reduction of diarrhea and the improvement of fecal scores was also reported for calves [17,21,36,37,38] without a clear mechanism behind these beneficial effects.

It is recognized that early life diarrhea episodes may decrease the richness and diversity of a developing foal’s gut microbiota [3,4], with a potential effect on the host immune system [39,40]. In light of the lack of significant effects of SCFP supplementation on FHD incidence, we did not analyze the fecal microbiota composition. Thus, it remains open whether SCFP supplementation affected the development of the foals’ gut microbiota. However, in a previous study, in which the same SCFP was used to supplement adult racehorses, no significant shift in fecal bacterial species could be demonstrated [32]. 

SCFP supplementation did not result in a significantly altered composition of circulating immune cell subsets 5–8 months after the final supplementation (Figure 3), although OLI foals tended to have higher numbers of circulating lymphocytes and in tendency a lower neutrophil/lymphocytes ratio (Figure 3c,i). This indicates that early life supplementation of foals with an SCFP does not affect the principal mechanisms of immune homoeostasis during the first months of life. However, the subtle differences before vaccination translated into drastic differences 24 h after vaccination: OLI foals responded with an increased number of nearly all tested immune cell subsets except CD4+ T cells (Figure 4 and Figure 5). The values in the PLA foals remained the same or increased only slightly, and none of the cell-count changes after vaccination of PLA foals reached significance. The changes (OLI) or absent changes (PLA) in circulating leukocyte numbers after vaccination were not correlated with negative side effects. Only in two PLA foals was the injection site slightly swollen, and one OLI foal had a slightly increased body temperature 24 h after vaccination. Thus, the findings strongly indicate that feeding foals with an SCFP during the first 30 days of life can have a profound long-term effect on the immune reactivity of the animals. 

Although not tested, the vaccination-induced changes in circulating immune cell numbers may indicate that the OLI foals released another spectrum and concentrations of mediators (e.g., cytokines, chemokines, or arachidonic acid metabolites), which either shape the release of cells from primary and/or secondary immune organs, or act on the emigration behavior of circulating immune cells from the blood stream. Such immediate vaccination responses were reported after vaccinations of humans with different vaccines [41,42,43], and an SCFP feeding-associated modulation of vaccination-induced mediator release was reported for bovines [30]. The altered response to vaccination after weaning clearly indicates that SCFP supplementation during the first 30 days of life can result in long-lasting effects on the immune reactivity of foals. Whether these observed changes translate into higher antibody titers against the vaccine antigens, robust responses to pathogens, or even overshooting and detrimental immune responses is currently unknown. Future trials should include the controlled challenge of SCFP-supplemented foals with real life pathogens.

The regulation and possible mechanisms behind this different immune reactivity to the first foal vaccination are currently unknown. Intrinsic host factors most likely play a minor role due to the equal age and sex distribution of the foals. Behavioral (e.g., exercise, acute and chronic physiological stress) and environmental factors (e.g., weather conditions, geographical location, season), as suggested by Lynn et al. [34], were the same for both foal groups. They received the identical basic feed, had the same grazing periods in the same area, and all foals were exposed to the same weaning stress and received the same vaccine at the same time after weaning. Since the gut microbiome has been shown to guide the response to vaccination [34,44,45], it has to be proven whether the early SCFP supplementation of foals has a profound impact on gut microbiome development. Although we previously refuted a strong prebiotic effect of the fed supplement in adult horses [32], this may turn out to be different in newborn foals. Another explanation for the observed differences between OLI and PLA foals could be the effects of resorbed postbiotics, which can act at the levels of bone marrow stem cells leading to long-term epigenetic modifications of such cells and their progeny [40,46,47,48]. 

Whether FHD itself has a long-term effect on immune responses later in life could not be addressed in this study since OLI and PLA foals were not equally distributed between diarrhea group 1 (DG1, 0–5 day of diarrhea) and diarrhea group 2 (DG2, ≥6 days of diarrhea) (Table 4). The diarrhea groups differed already significantly in the composition of circulating blood cells on the day of vaccination (Figure S1). DG2 foals, for instance, presented with significantly lower numbers of lymphocytes, CD8+ T cells, and MHC-II+/CD21− cells. This might suggest an FHD-induced effect on long-term blood immune cell homeostasis. However, DG1 and DG2 foals did not differ in their responses to vaccination (Figure S2), most likely due to the unequal representation of OLI and PLA foals in the diarrhea groups. 

## 5. Conclusions

The feeding of newborn foals with a pre-/postbiotic SCFP did not alter significantly the incidence and the severity of foal heat diarrhea. However, SCFP supplementation during the first 30 days of life resulted in long-lasting effects on the immune reactivity of foals. Such an *S. cerevisiae* fermentation product-induced immune modulation may not only affect early responses to vaccination but also initial reactions after pathogen contact. This indicates that ingredients of the SCPF and not an altered diarrhea frequency are responsible for the observed effects. Conclusions on diarrhea-mediated long-term effects can be drawn in future when similarly fed and raised FHD-negative and -positive foals are compared in their response to an immunological challenge. 

## Figures and Tables

**Table 1:** Ingredients of feed supplements.

Ingredient	OLI ^1^ (10 g)	PLA ^2^ (10 g)
Tocopherol extract	0.045 g	0.045 g
Coconut oil (rapeseed oil)	-	0.3 g
Vitamin C	0.292 g	0.292 g
Dextrose	-	2.00 g
Corn cob meal	-	1.5 g
Linseed cake	-	1.5 g
Microcrystalline cellulose	-	2.00 g
Minerals	0.063 g	2.363 g
Inactivated yeasts	9.6 g	-

^1^ OLI, Olimond BB; ^2^ PLA, placebo. Each foal received 10 g/day. The composition was chosen to mimic the color, the texture, and the vitamin content of the SCFP.

**Table 2:** Colostrum supply and growth characteristics of foals.

		Group 1 (OLI) (*n* = 11)	Group 2 (PLA) (*n* = 10)	*p* ^c^
Brix (%)		23.5 (17–32)	25.0 (22–33)	0.1923
Serum protein (g/dL) ^a^		6.0 (4.6–6.6)	6.4 (5.6–7.2)	0.3567
Height (cm) ^b^	d2	105.0 ± 1.0	104.0 ± 4.25	0.6690
	d15	110.0 ± 2.5	111.0 ± 2.5	0.5685
	d30	115.0 ± 2.5	115.5 ± 5.25	0.5708
Weight (kg) ^b^	d2	59.6 ± 7.45	58.4 ± 8.55	0.6985
	d15	82.7 ± 8.35	80.9 ± 10.925	0.8602
	d30	101.0 ± 12.85	98.1 ± 16.25	0.5974

(a) Serum total protein was determined on day 2; (b) median ± interquartile range (IQR); (c) *p* values were determined by Wilcoxon two-sample test.

**Table 3:** Days with diarrhea and severity of foal diarrhea during days 2–30 after birth.

	Group 1 (OLI) (*n* = 11)	Group 2 (PLA) (*n* = 10)	*p*
Diarrhea days ^a^	2.0 ± 4.5	6.0 ± 5.5	0.1347
Diarrhea severity ^b^	8.0 ± 16.0	23.5 ± 21.25	0.1288

(a) Number of days (median ± IQR) with feces score of 1 (watery) or 2 (thin mushy); (b) feces scores of all 29 days were transformed into severity points (median ± IQR of severity point sum).

**Table 4:** Number of foals in diarrhea severity groups.

Diarrhea Group (DG) ^a^	Group 1 (OLI) (*n* = 11)	Group 2 (PLA) (*n* = 10)
DG1	8	4
DG2	3	6

(a) DG1: 0–5 days with feces score 1 or 2; DG2: 6–8 days with feces score 1 or 2; Fisher’s exact test was used to determine whether there were differences in the number of foals in DG1 or DG2, when the foals were fed either the supplement or the placebo (*p* = 0.1984).

## Data Availability

None of the data was deposited in an official repository. The data that support the study findings are available upon reasonable request.

## Supplementary Materials

The following supporting information can be downloaded at: https://www.mdpi.com/article/10.3390/ani14060960/s1, Figure S1: Comparison between diarrhea groups (DG1 = diarrhea group 1, 0–5 days; DG2 = diarrhea group 2, 6–8 days) before vaccination in the age of 6–9 months; Figure S2: Comparison of the early response of foals after vaccination between diarrhea groups (DG1, DG2). Figure S3: Fluorescence compensation settings; Table S1: Average age and parity of mares; Table S2: Feces scoring system and diarrhea severity; Table S3: Vaccination groups; Table S4: Foal appearance and rectal temperatures.
