# Supplementary material for: Supplementation of Foals with a Saccharomyces cerevisiae Fermentation Product Alters the Early Response to Vaccination
Source: Animals (Basel). 2024 Mar 20;14(6):960. doi: 10.3390/ani14060960 (PMC10967450; doi:10.3390/ani14060960)
Supplement: Supplementary file 1 [file animals-14-00960-s001.zip › animals-2883850-supplementary.pdf]

Table S1. Average age and parity of mares.

	N	Mean Age (Min-Max)	Mean Parity (Min-Max)
**Group (OLI)**	11[1]	11 (4 - 18)	3.45 (0 - 6)
**Group (PLA)**	11[2]	11 (4 - 18)	3.64 (0 - 6)

[1]one foal had to be euthanized after intensive colic treatment before weaning; [2]one foal died 3 days after birth

Table S2. Feces scoring system and diarrhea severity.

Score	Feces consistence	Severity points
1	Watery	4
2	Thin mushy	3
3	Mushy pasty	1
4	Formed	0

Score 1 + 2 were classified as diarrhea.

Table S3. Vaccination groups.

A) Vaccination Oct 4th 2022			B) Vaccination Nov 8th 2022		
**Foal**	**OLI/ PLA[1]**	**Age (months)**	**Foal**	**OLI/ PLA**	**Age (months)**
1	OLI	8	8	OLI	9
2	OLI	8	10	PLA	8
3	PLA	8	13	PLA	7
4	PLA	8	14	OLI	7
5	PLA	8	15	PLA	7
6	OLI	8	16	PLA	6
7	OLI	8	17	OLI	6
9	PLA	7	19	OLI	6
11	PLA	6	20	OLI	6
12	OLI	6	22	PLA	6
Mean ± s		7.5 ± 0.85			6.8 ± 1.03

1) Foals were fed a SCFP (OLI) or a placebo (PLA) between day 2 until day 30 post partum.

Table S4. Foal appearance and rectal temperatures.

Foal	Day	Rectal body temp.	Coat	Nutritional condition	Feces	Limbs	Behavior[1]
1	2	38.5	plushy	good	formed	normal	normal
	15	38.2	smooth	well nourished	formed	normal	twitchy
	30	38.2	smooth	well nourished	formed	normal	normal
2	2	38.4	normal	skinny	formed	toe-wide	normal
	15	38.3	plushy dull	good	formed	normal	normal
	30	38.2	plushy dull	good	formed	nearly normal	normal
3	2	38.3	plushy	good	formed	normal	normal
	15	38.4	smooth	good	formed	normal	pithy
	30	38.2	plushy	well nourished	formed	normal	normal
4	2	38.3	plushy	good	formed	soft pastern	weary
	15	38.4	normal	good	soft	soft	sleeps a lot
	30	38.2	dense	good	formed	improved	weary
5	2	38.1	smooth	good	formed	normal	vital
	15	38.8	smooth	good	formed	malposition	weary
	30	38.2	dissimilar	good	formed	nearly normal	calm
6	2	38.4	plushy	good	formed	hind legs round	normal
	15	38.3	firm	good	formed	normal	sensible
	30	38.0	dense	burly	formed	normal	explosive
7	2	38.4	normal	good	formed	hind legs minimal soft	normal
	15	38.5	plushy dull	good	formed	toe in the air	agile
	30	38.2	dull	good	formed	still remains	calm, stubborn
8	2	38.3	smooth	good	formed	normal	pithy
	15	38.4	smooth	good	watery	normal	normal
	30	38.2	dense	good	formed	normal	normal
9	2	38.4	normal	good	formed	normal	bright
	15	38.1	normal	good	formed	normal	powerful
	30	38.1	smooth dense	well nourished	formed	normal	powerful
10	2	38.4	dense	well nourished	formed	hind legs soft	calm
	15	38.4	dull	skinny	formed	normal	normal
	30	38.2	smooth	good	formed	normal	strong, calm
11	2	38.5	smooth	good	formed	a bit steep	strong
	15	38.3	smooth dense	good	formed	normal	strong
	30	38.3	smooth dense	good, burly	formed	normal	strong, calm
12	2	38.6	normal	good	formed	hind right leg windswept	calm
	15	38.1	shaggy	good	thin mushy	normal	strong
	30	38.1	shaggy	good	formed	normal	strong

Table S4. Continued.

13	2	38.4	smooth	tender	formed	normal	normal
	15	38.2	dense	burly	formed	normal	strong
	30	38.2	dense, dull	burly	formed	normal	normal
14	2	38.6	normal	good	formed	normal	shy
	15	38.2	dense, dull	good	formed	normal	twitchy
	30	-	dense, dull	good	formed	normal	calm
15	2	38.7	dense, smooth	good	formed	steep, esp. hind legs	normal
	15	38.2	smooth	good	formed	normal	anxious
	30	38.2	smooth	good	formed	normal	normal
16	2	38.7	smooth	skinny	formed	normal	normal
	15	38.2	smooth	good	watery	normal	normal
	30	38.0	dull	good	formed	normal	normal
17	2	38.4	short	good	formed	hind legs soft	calm
	15	38.3	short	good	watery – thin mushy	hind legs round	paranoid
	30	38.2	short	good	formed	normal	normal
18	2	38.4	dense	good	formed	hind legs soft	calm, shy
	15	38.3	dense, long	good	formed	hind legs soft	calm, shy
	30	38.1	dense, smooth	good	formed	hind legs round	calm, shy
19	2	38.7	slightly open	good	formed	hind legs soft	normal
	15	38.4	dense, smooth	good	formed	normal	calm
	30	-	dense, smooth	good	formed	normal	calm
20	2	38.6	dense	good	formed	long, soft pastern	calm
	15	-	dense	good	formed	significantly higher	calm
	30	-	dense	good	formed	normal	calm
22	2	-	short, dense	good	formed	flexor tendon contracture	calm
	15	-	short, dense	slim	formed	long shoulder hind legs soft	fast, calm
	30	-	dense, smooth	good	formed	normal	calm

1) Strong: strong character, calm in head, good to handle. Powerful: strong but more difficult to handle. Explosive: very powerful and nearly not to handle. Pithy: robust, fresh, a little stubborn. Twitchy: tensed, jumpy on contact.

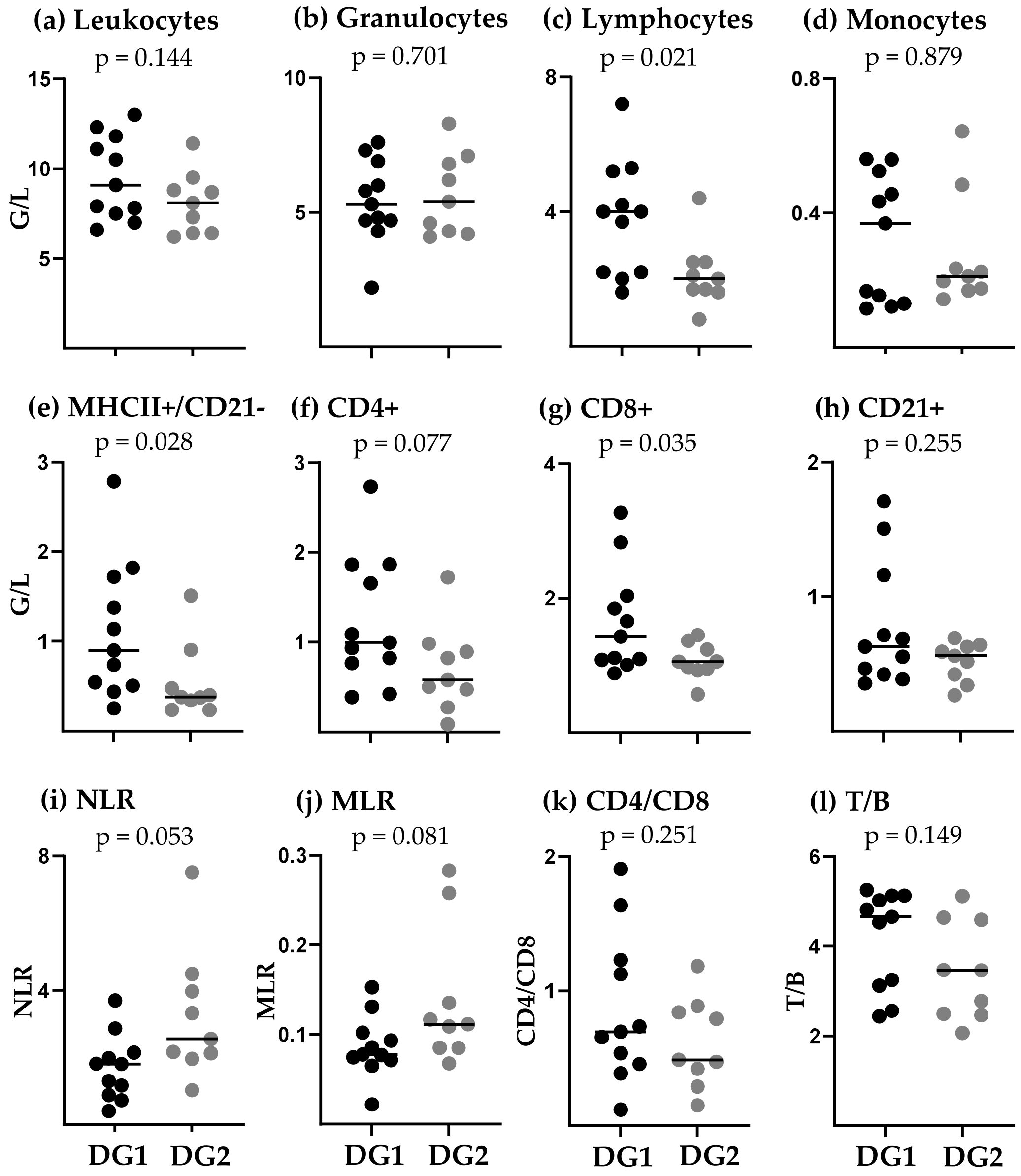

Figure S1. Numbers of major leukocyte populations, leukocyte subpopulations (G/L, giga/liter) and ratios between leukocytes in blood of foals before vaccination at the age of 6-9 months. Foals were grouped according to the severity and duration of foal heat diarrhea (DG1, diarrhea group 1, n = 11, 0-5 days of diarrhea; DG2, diarrhea group 2, n = 9, 6-8 days of diarrhea). Total leukocyte numbers (a) were determined in a counting chamber and were used to calculate absolute numbers of leukocyte subpopulations (b-h) after flow cytometric measurement of their fraction among leukocytes (e: MHC-II+ and CD21- lymphocytes; f: CD4+ T cells; g: CD8+ T cells; h: CD21+ B cells). i-l: Ratios between neutrophils and lymphocytes (i: NLR), monocytes and lymphocytes (j: MLR), CD4+ and CD8+ T cells (k: CD4/CD8), T (sum of CD4+ and CD8+ T cells) and B cells (l: T/B). P values were determined by unpaired t-test or Wilcoxon two-sample test.

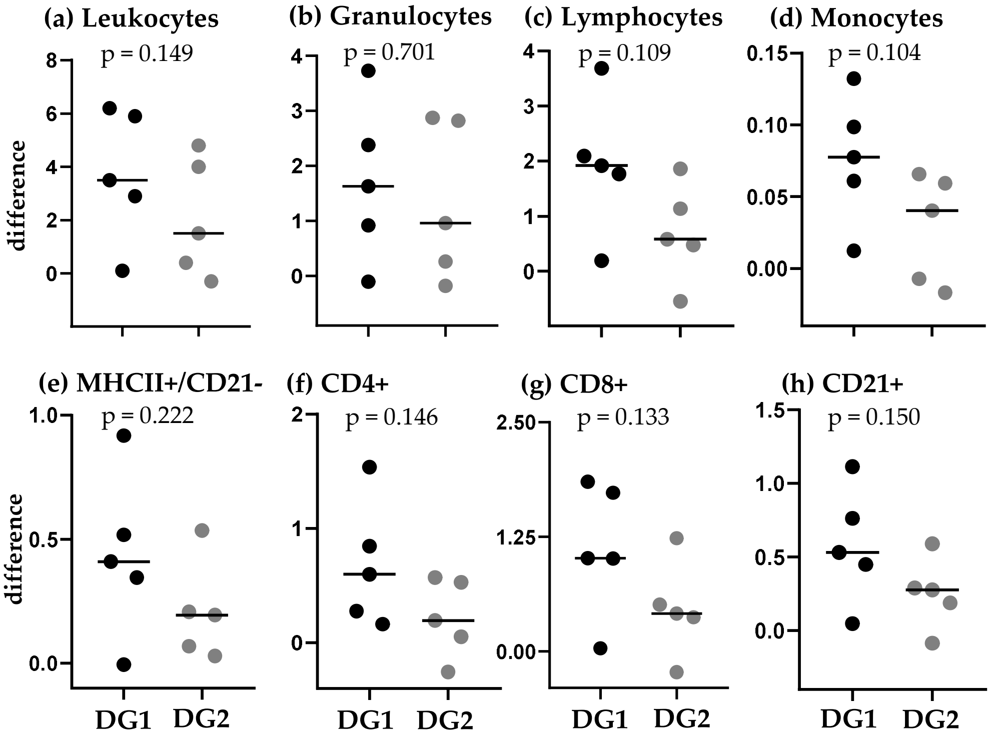

Figure S2. Changes in numbers of circulating major leukocyte populations and leukocyte subpopulations within 24h after vaccination of foals. Foals were grouped according to the severity and duration of foal heat diarrhea (DG1, diarrhea group 1, n = 5, 0-5 days of diarrhea; DG2, diarrhea group 2, n = 5, 6-8 days of diarrhea). Total leukocyte numbers (a) were determined in a counting chamber and were used to calculate absolute numbers of leukocyte subpopulations (b-h) after flow cytometric measurement of their fraction among leukocytes. The values show the difference between the absolute numbers number (giga/liter blood) determined after and before vaccination (e: MHC-II+ and CD21- lymphocytes; f: CD4+ T cells; g: CD8+ T cells; h: CD21+ B cells). P values were determined by unpaired t-test or Wilcoxon two-sample test.

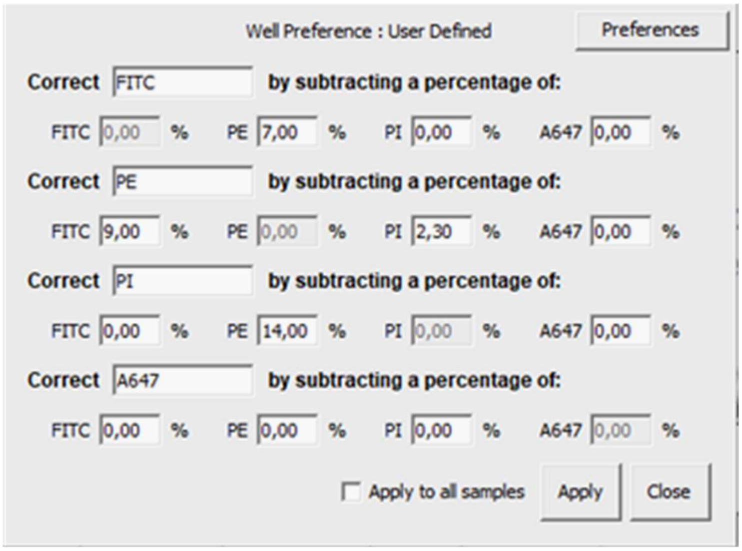

Figure S3. Fluorescence compensation settings (BD Accuri C6 plus Software). Compensation was applied after acquisition of the samples.
